# Listening to Patients’ Voices on the Use of AI in Health Care: Cross-Sectional Study

**DOI:** 10.2196/77501

**Published:** 2025-12-05

**Authors:** Ranganathan Chandrasekaran, Lavanya Takale, Evangelos Moustakas

**Affiliations:** 1 Department of Information & Decision Sciences, University of Illinois Chicago, 2428 Univ Hall, 601 S Morgan Street, Chicago, IL, 60607, United States, 1 3129962847; 2 Department of Communication and Media, Canadian University of Dubai, Dubai, United Arab Emirates

**Keywords:** patient attitudes, artificial Intelligence, technology acceptance, responsible AI, survey

## Abstract

**Background:**

Artificial intelligence (AI) holds great promise in transforming health care delivery. However, successful implementation of AI projects in health care depends on patients’ acceptance and trust. There is limited empirical research examining public perceptions, particularly the use of personal health data in AI applications in health care.

**Objective:**

This study examined public knowledge and comfort levels with AI use in health care, including use of personal health data with and without consent, and assessed how sociodemographic factors, digital literacy, and health conditions influence these perceptions.

**Methods:**

We analyzed data from 6904 Canadian adults who participated in the 2023 Canadian Digital Health Survey. AI-related knowledge and comfort levels were measured using ordinal scales. Sociodemographic characteristics, digital health literacy, and self-reported chronic health conditions were included as predictors. Ordinal logistic regression models were used to assess associations between these factors and AI-related attitudes.

**Results:**

A majority of 2919 (42.3%) reported moderate knowledge of AI; only 7.8% (542) described themselves as very knowledgeable. Overall, 44.6% were comfortable with AI use in health care, increasing to 64.7% when personal health data were used with consent but decreasing when used without consent (52.6% uncomfortable). Respondents were most comfortable with AI use for epidemic tracking and workflow management and less for clinical tasks. Fully weighted ordinal logistic regression models indicated that men (odds ratio [OR]=1.57, *P*<.001), noncitizens (OR=1.71, *P*<.001), higher-income respondents (OR=1.29, *P*<.001), those with graduate education (OR=1.43, *P*<.001), higher digital health literacy (OR=1.08, *P*<.001), and more chronic conditions (OR=1.08, *P*<.001) exhibited greater odds of reporting higher AI knowledge. For comfort with AI use in health care, those aged 65+ years (OR=1.47, *P*<.001), men (OR=1.50, *P*<.001), noncitizens (OR=1.49, *P*<.001), higher-income respondents (OR=1.21, *P*<.001), and those with higher digital health literacy (OR=1.06, *P*<.001) or more chronic conditions (OR=1.04, *P*=.04) exhibited greater comfort. Lower-income (OR=0.87, *P*=.03) and White respondents (OR=0.77, *P*<.001) reported lower comfort levels. For comfort with using personal health data in AI with consent, adults aged 35‐54 years (OR=0.72, *P*<.001) were less comfortable than those aged 16‐24 years. Men (OR=1.39, *P*<.001), higher-income respondents (OR=1.16, *P*=.01), and those with higher digital health literacy (OR=1.05, *P*<.001) or more chronic conditions (OR=1.07, *P*<.001) showed greater comfort; White (OR=0.78, *P*<.001), other racial groups (OR=0.77, *P*=.03), and lower-income respondents were less comfortable (OR=0.83, *P*=.01). For comfort with using personal health data in AI without consent, men (OR=1.56, *P*<.001), noncitizens (OR=1.28, *P*=.03), and those with higher digital health literacy (OR=1.04, *P*<.001) exhibited greater comfort. Lower-income respondents (OR=0.86, *P*=.02), adults aged 35‐54 years (OR=0.73, *P*<.001) or 55‐64 years (OR=0.77, *P*=.01), and White (OR=0.69, *P*<.001) and Black or African-origin (OR=0.71, *P*=.02) respondents reported lower comfort levels.

**Conclusions:**

The findings point to enhancing transparent policies, digital literacy, and ethical data governance as key to increasing public trust in AI-driven health care.

## Introduction

Artificial intelligence (AI) in health care broadly refers to the use of advanced computational techniques and algorithms, including machine learning, deep learning, natural language processing, large language and image models, and computer vision to extract insights from complex medical data and enhance clinical decision-making [1,2]. By augmenting human expertise with data-driven insights, AI has the potential to revolutionize multiple aspects of health care delivery, from early disease detection [3,4] and drug discovery [5,6] to improving operational efficiency and resource allocation in health care systems [7]. Advanced generative AI models can help analyze a variety of digital content, including clinical images, videos, text, and audio, as well as clinical data from electronic health records [8]. The global AI in health care market is projected to grow at a compound annual growth rate of 36.83% from 2024 to 2034, increasing from US $26.69 billion to US $613.81 billion [9], signaling a significant shift in how health care is delivered and managed. This rapid integration of AI into clinical practice has generated both excitement and concern among health care professionals, policymakers, and patients. As AI becomes more prevalent in clinical settings, there is a growing concern about its unintended consequences. AI could deepen the digital divide and increase disparities, especially among older adults, low-income groups, and rural communities [10]. Further, the ethical and regulatory challenges surrounding the use of AI in health care continue to be widely debated [11].

While the transformative potential of AI in health care is well understood, its expanded adoption raises important questions about its impact on patient care, equity, and ethics. Patients, as the ultimate beneficiaries of AI-driven health care solutions, play a critical role in shaping how AI technologies are designed, implemented, and trusted. However, patient perspectives on AI remain underexplored. Understanding these perspectives is important for many reasons. First, it ensures patient trust and acceptance, which are essential for successful implementation of AI-based health care solutions [12]. Second, it helps address ethical concerns and potential biases in AI algorithms, which could lead to unequal access to care or diagnostic errors [13]. Third, a good understanding of patients’ views on AI allows for tailoring AI solutions that align with patient needs and preferences, ultimately improving health outcomes and satisfaction [14].

Despite the importance of understanding patient perspectives on AI use in clinical practice, research in this area remains limited. While several studies have explored health care professionals’ attitudes toward AI [15-22], comparatively little attention has been given to patients’ attitudes and concerns. Some studies have reported a generally positive patient attitude toward the use of AI in health care [23,24], though they also highlight concerns about privacy and control over personal health data. Other studies have documented patient resistance [25], distrust in AI [26], and a preference for restricting AI use to nonclinical tasks such as administrative or scheduling functions [27]. In addition, studies have also identified patient apprehensions due to perceived safety risks, threats to patient autonomy, potential increases in health care costs, algorithmic biases, and data security issues [28].

**Figure 1.**
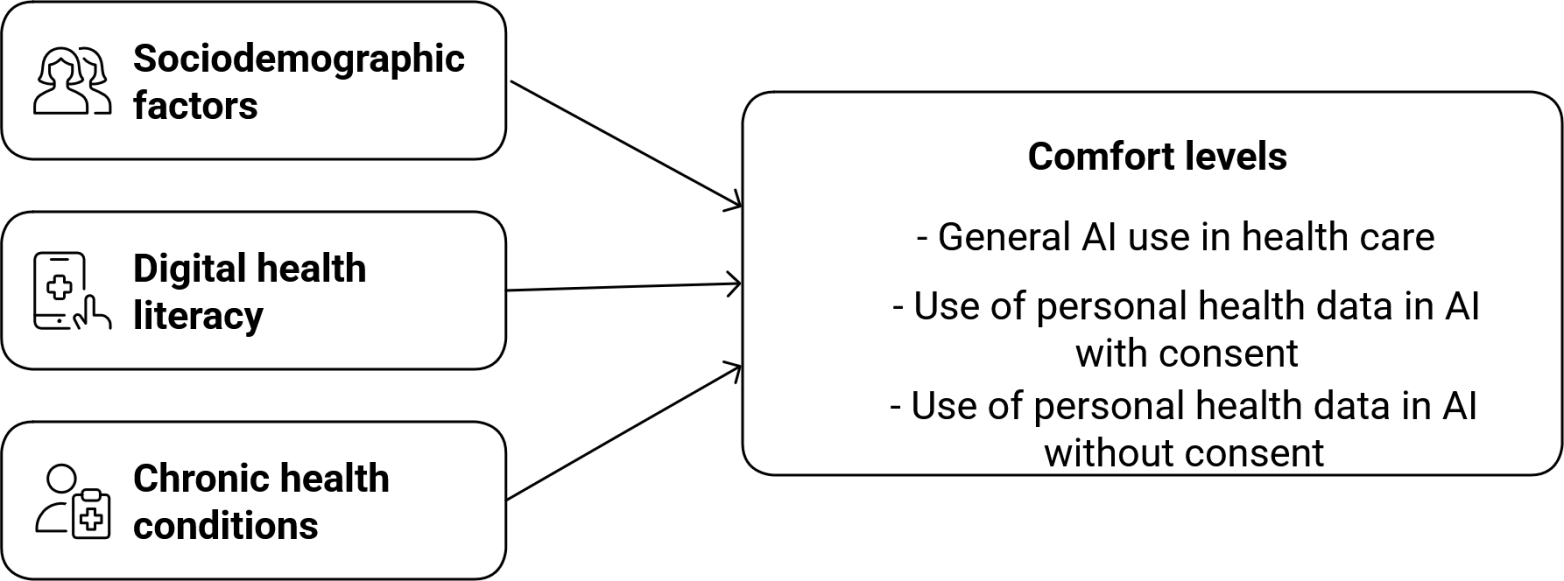
Research framework. AI: artificial intelligence.

This study examines patients’ knowledge levels regarding AI in health care, their comfort with AI use across clinical applications, and their attitudes toward the use of personal health information for AI purposes. Our research framework is shown in Figure 1. We also explore how sociodemographic characteristics, digital health literacy, and health conditions are associated with patient attitudes and comfort levels. Specifically, we address two research questions: (1) What are the levels of public knowledge and comfort with AI in health care, including the use of personal health data with and without consent? and (2) How do sociodemographic factors, digital health literacy, and chronic health conditions influence these attitudes? By addressing these questions, this study aims to fill critical gaps in understanding patient perspectives and to inform ethical, equitable, and effective implementation of AI solutions in health care.

## Methods

### Dataset

Data used in this study were obtained from the 2023 Canadian Digital Health Survey (CDHS) that was commissioned by Canada Health Infoway to assess Canadians’ experiences and perceptions regarding digital health services, including the use of AI in health care [29,30]. The web-based survey was administered by Leger, one of Canada’s leading market research firms, between November 28 and December 28, 2023. Participants, aged 16 years and older, were recruited from Leger Opinion’s nationally representative online panel using computer-assisted web interviewing technology. A total of 10,130 respondents participated in the survey, which was available in both English and French.

### Ethical Considerations

The 2023 Canadian Digital Health Survey obtained informed consent from its 10,130 participants and adhered to the public opinion research standards of the Canadian Research and Insights Council and the global ESOMAR (European Society for Opinion and Marketing Research) network to ensure methodological rigor and data quality [29,31]. Information about respondents was deidentified and anonymized to protect privacy and confidentiality. Patients provided consent for data collection and evaluation.

### Variables

The survey assessed four key variables related to patients’ perceptions of AI in health care using 4-point ordinal scales. To evaluate participants’ understanding of AI, they were asked to rate their knowledge on a scale from 1 (not at all knowledgeable) to 4 (very knowledgeable). The question “How comfortable are you with AI being used as a tool in health care?” was used to assess participants’ comfort level on a scale ranging from 1 (very uncomfortable) to 4 (very comfortable). A similar approach was used to assess attitudes toward the use of personal health data in AI research. Participants were asked how comfortable they felt about scientists using their personal health data for AI research when informed consent was provided, using the same 4-point scale. To examine privacy concerns, the survey also asked about comfort levels regarding AI research using deidentified health data without explicit consent. Participants were also asked about their comfort levels in applying AI in 7 areas: monitoring and predicting health conditions, decision support for health care professionals, precision medicine, drug and vaccine development, disease monitoring at home, tracking epidemics, and optimizing health care workflows.

Participants were asked to self-report any serious or chronic health conditions diagnosed by a health professional. The survey defined chronic illness as a condition expected to last, or already lasting, 6 months or more. Respondents could choose from a predefined list of 15 chronic conditions: chronic pain, cancer, diabetes, cardiovascular disease, Alzheimer’s disease, developmental disabilities, obesity, mental health conditions, and physical or sensory disabilities. Additionally, participants had the option to specify any other chronic illness not listed or indicate no chronic illness. We calculated a composite score representing the total number of chronic conditions reported by each respondent.

Digital health literacy was assessed using 8 items from eHealth Literacy Scale [32], which measures the ability to find, evaluate, and use health information on the internet. Items were rated on a 5-point Likert scale (1=strongly disagree to 5=strongly agree). After confirming their convergence and reliability using exploratory principal component analysis with varimax rotation (which extracted one factor, confirming unidimensionality) and Cronbach α (0.934), responses were summed to create a digital health literacy score, reflecting a respondent’s overall proficiency in navigating and utilizing online health resources. The following sociodemographic variables were also captured in the survey: age, sex, annual household income, citizenship, race, educational attainment, and employment status.

### Analytic Sample and Nonresponse Bias

CDHS collected data from 10,130 Canadian adults on a broad range of topics related to digital health. Given this study’s focus on AI use in health care, only 6904 respondents who provided complete responses to AI-related questions were included in the analytic sample.

To assess potential selection or nonresponse bias, *χ*^2^ tests were conducted to compare included and excluded respondents across all the sociodemographic variables: age, sex, income, education, race, employment, and citizenship. The χ^2^ tests revealed significant differences between the overall respondents and our analytic sample across five variables (age, sex, employment, education, and income; *P*<.05), with our analytic sample overrepresenting males (53.2% vs 48.6%), individuals aged 25‐54 years (51.8% vs 48.6%), higher household incomes (35.7% above CAD 100,000 vs 33.6%), higher education levels (eg, graduate college or above: 39.7% vs 37.4%), and employed respondents (61.6% vs 58.6%).

To address this bias, we derived inverse probability weights (IPW) to adjust for the likelihood of inclusion in the analytic sample. The IPW values were estimated using a logistic regression model predicting inclusion based on sociodemographic variables. These weights were then combined with the original CDHS survey design weights (which account for sampling and nonresponse at the national level) to create a composite total weight. This combined weighting approach ensured that both survey design and sample selection bias were accounted for in all weighted analyses.

### Statistical Analysis

All statistical analyses were conducted using STATA 18 software. Descriptive statistics summarized respondent characteristics. To evaluate potential selection bias, the IPW was derived as described above.

Ordinal logistic regression models were estimated for four AI-related attitudinal outcomes: (1) knowledge of AI in health care, (2) comfort with use of AI in health care, (3) comfort with use of personal health data for AI with consent, and (4) comfort with use of personal health data for AI without consent. Each model was estimated three ways: unweighted, nonresponse-adjusted weighted (IPW), and fully weighted (combining IPW and CDHS-provided survey design weights) to evaluate robustness. All weighted models were estimated using survey (svy) commands in STATA to account for the complex survey design. Potential multicollinearity among predictors was assessed using Cramer’s V for categorical variables and variance inflation factors from proxy linear regressions.

## Results

The demographic profile of survey respondents is presented in Table 1. Our dataset had the majority of respondents aged 35‐54 years (2412, 34.94%), followed by those aged 65+ years (1585, 22.96%) and 55‐64 years (1211, 17.54%). There was a slight majority of male respondents (53.2%, 3673), with female respondents comprising 46.8% (3231) of the sample. Regarding household income, 4699 (68.06%) reported earnings of CAD 60,000 or more, while 2205 (31.94%) earned less. The majority were Canadian citizens (6581, 95.32%), with noncitizens (323, 4.68%) forming a smaller proportion. Racially, the sample had predominantly White respondents (5104, 73.93%), followed by Asian-origin (972, 14.08%), other (575, 8.33%), and Black or African-origin (253, 3.66%) respondents. Education levels varied, with most having at least some college education (2831, 41.01%) or a graduate degree (2738, 39.66%). Fewer had a high school diploma (1174, 17%) or less than high school education (161, 2.33%). Employment status showed that 4253 (61.6%) were employed.

**Table 1.** Sociodemographic characteristics of respondents (n=6904).

Demographic variable and category	n (%)
Age group (y)	
16‐24	527 (7.63)
25‐34	1169 (16.93)
35‐54	2412 (34.94)
55‐64	1211 (17.54)
65+	1585 (22.96)
Sex	
Female	3231 (46.8)
Male	3673 (53.2)
Household income (CAD)^a^	
<60,000	2205 (31.94)
60,000‐100,000	2237 (32.4)
>100,000	2462 (35.66)
Citizenship	
Citizen	6581 (95.32)
Noncitizen	323 (4.68)
Race	
Asian origin	972 (14.08)
Black/African origin	253 (3.66)
Other	575 (8.33)
White	5104 (73.93)
Education	
Less than high school	161 (2.33)
High school	1174 (17)
College level	2831 (41.01)
Graduate college or above	2738 (39.66)
Employment	
Employed	4253 (61.6)
Unemployed	2651 (38.4)

a A currency exchange rate of CAD $1=approximately US $0.72 is applicable.

**Figure 2.**
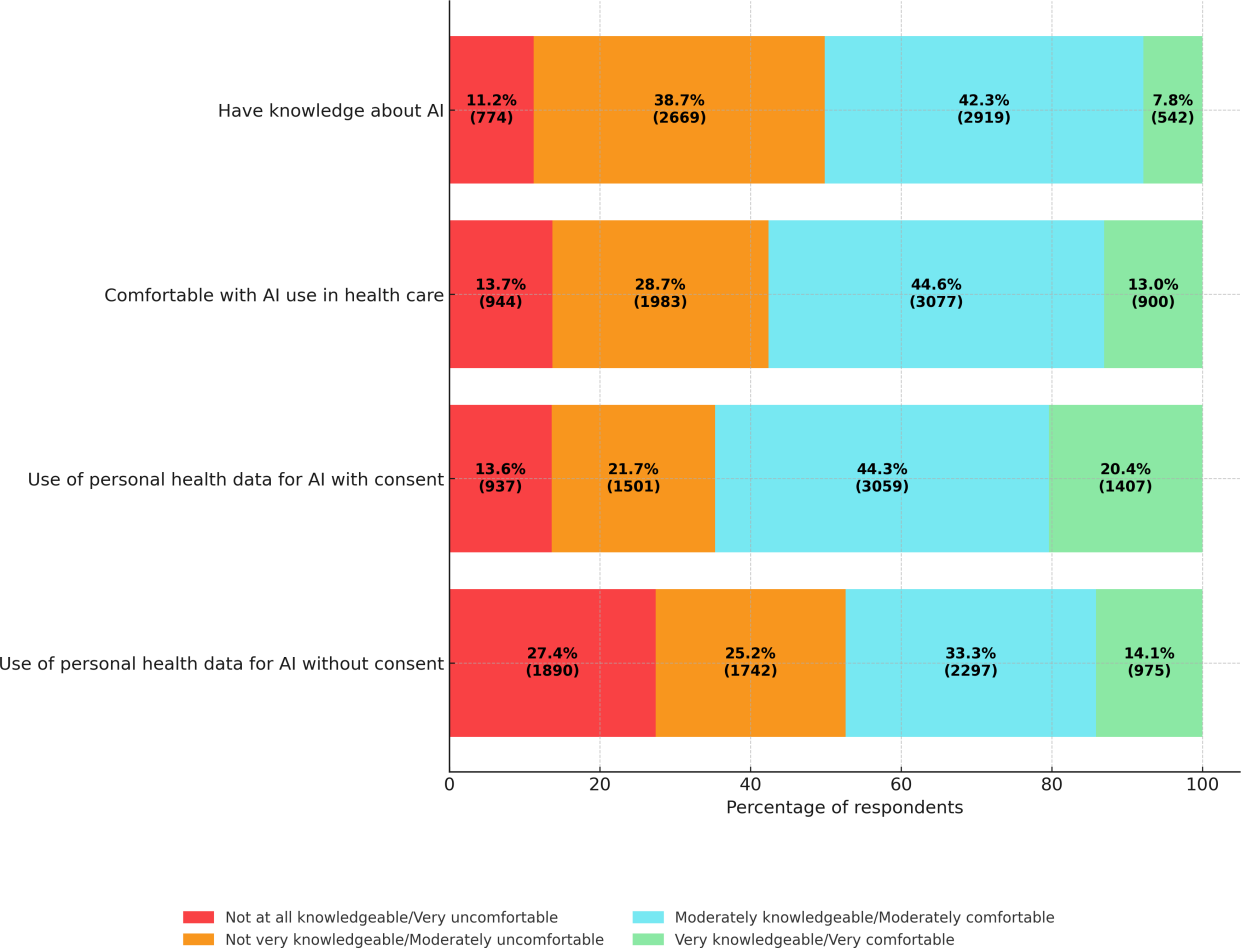
Distribution of self-reported AI knowledge and comfort levels among Canadian adults (n=6904) in the 2023 Canadian Digital Health Survey. AI: artificial intelligence.

Our analysis found varying levels of knowledge levels and comfort regarding AI use in health care among respondents (Figure 2). While a majority (2919, 42.3%) reported being moderately knowledgeable about AI, only 7.8% (542) considered themselves very knowledgeable. Conversely, nearly half of the respondents (49.9%) considered themselves less knowledgeable, with 38.7% (2669) reporting they were “not very knowledgeable” and 11.2% (774) reporting “not at all knowledgeable.”

**Figure 3.**
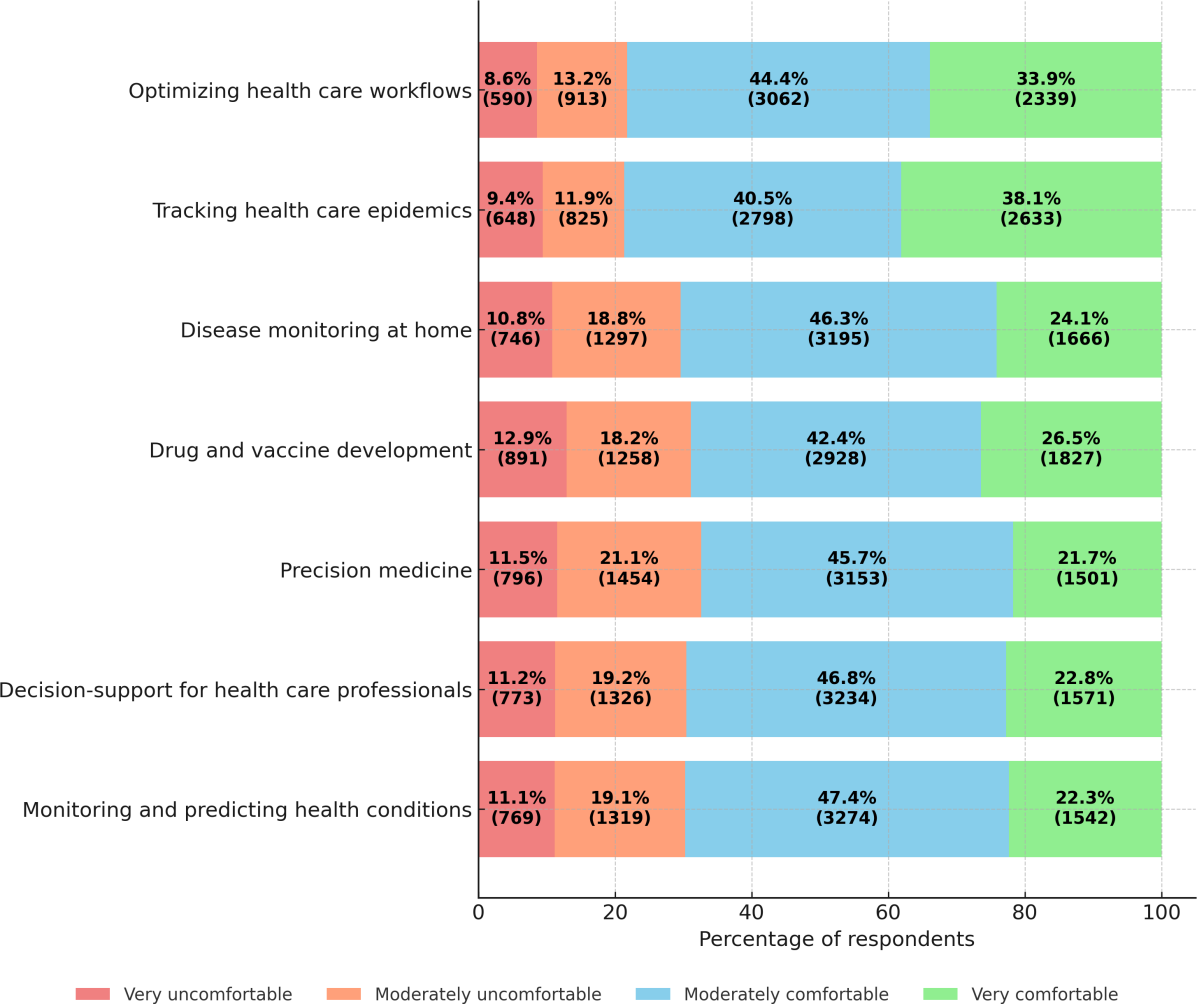
Comfort levels with AI applications in specific health care areas among Canadian adults (n=6904) in the 2023 Canadian Digital Health Survey. AI: artificial intelligence.

When it comes to AI use in health care, 44.6% (3077) of respondents reported being moderately comfortable, while 42.4% (2927) expressed some level of discomfort. Comfort levels increased when AI involved use of personal health data under informed consent, with 64.7% (4466, moderately or very comfortable) supporting such AI use. However, comfort levels declined when AI research used deidentified data without consent, with only 47.4% (3272) reporting comfort and 52.6% (3632) expressing discomfort. When asked about comfort levels pertaining to AI use in various health care areas (Figure 3), moderate comfort levels (40%‐47%) were observed across all areas. However, respondents expressed relatively greater support for AI use in tracking epidemics and optimizing health care workflows, where a higher proportion of respondents felt “very comfortable” compared to other areas.

Table 2 presents results from the fully weighted ordinal logistic regression models assessing associations between respondents’ self-reported levels of knowledge about AI and their sociodemographic characteristics, digital health literacy, and health conditions. These results were consistent with those from the unweighted and nonresponse-adjusted models, showing similar effect sizes and significance patterns (Multimedia Appendix 1).

**Table 2.** Ordinal regression results (fully weighted): association between AI knowledge levels and sociodemographic factors, digital health literacy, and Health Conditions in 2023 Canadian Digital Health Survey.

Predictors and category	OR (95% CI)	*P* value
Age group (ref: 16‐24 y)		
25‐34 y	0.69 (0.54‐0.87)	<.001
35‐54 y	0.59 (0.47‐0.73)	<.001
55‐64 y	0.44 (0.35‐0.56)	<.001
65+ y	0.39 (0.31‐0.49)	<.001
Sex (ref: female)		
Male	1.57 (1.42‐1.73)	<.001
Household income (ref: CAD 60,000‐100,000)		
CAD >100,000	1.29 (1.14‐1.45)	<.001
CAD <60,000	1.07 (0.94‐1.22)	.34
Citizenship (ref: citizen)		
Noncitizen	1.71 (1.32‐2.21)	<.001
Race (ref: Asian)		
Black/African origin	1.00 (0.74‐1.36)	.99
Other	0.93 (0.72‐1.20)	.56
White	0.79 (0.68‐0.93)	<.001
Education (ref: college level)		
Graduate college and higher	1.43 (1.27‐1.60)	<.001
High school	1.03 (0.89‐1.20)	.70
<High school	0.97 (0.66‐1.41)	.87
Employment (ref: employed)		
Unemployed	1.09 (0.95‐1.24)	.21
Digital health literacy	1.08 (1.07‐1.09)	<.001
Number of chronic conditions	1.08 (1.03‐1.12)	<.001

Age was a significant predictor, with respondents in older age groups exhibiting lower odds of having higher AI knowledge compared to those aged 16‐24 years: 25‐34 years (odds ratio [OR] 0.69, 95% CI 0.54‐0.87; *P*<.001), 35‐54 years (OR 0.59, 95% CI 0.47‐0.73; *P*<.001), 55‐64 years (OR 0.44, 95% CI 0.35‐0.56; *P*<.001), and 65+ years (OR 0.39, 95% CI 0.31‐0.49; *P*<.001). Men were significantly more likely to report higher AI knowledge than women (OR 1.57, 95% CI 1.42‐1.73; *P*<.001).

Among socioeconomic factors, those with higher annual household incomes (CAD >100,000) exhibited higher odds for greater AI knowledge (OR 1.29, 95% CI 1.14‐1.45; *P*<.001), while those with lower incomes (CAD <$60,000) showed no significant difference (OR 1.07, 95% CI 0.94‐1.22; *P*=.34). Noncitizens exhibited higher AI knowledge levels (OR 1.71, 95% CI 1.32‐2.21; *P*<.001) compared to citizens. Race also was a significant factor, with White respondents exhibiting lower odds of AI knowledge (OR 0.79, 95% CI 0.68‐0.93; *P*<.001) relative to Asian-origin respondents, while differences for Black or African-origin and Other groups were not statistically significant.

Education was another key predictor, with graduates showing significantly higher AI knowledge (OR 1.43, 95% CI 1.27‐1.60; *P*<.001) compared to those with a college-level education, while respondents with only a high school education or less showed no significant difference. Employment status was not significantly associated with the odds of reporting higher AI knowledge.

Higher digital health literacy was strongly associated with increased AI knowledge (OR 1.08, 95% CI 1.07‐1.09; *P*<.001). Additionally, respondents with more chronic health conditions had higher odds of reporting greater AI knowledge (OR 1.08, 95% CI 1.03‐1.12; *P*<.001), suggesting that health experiences may influence awareness of AI applications.

Table 3 presents results from the fully weighted ordinal logistic regression models, each examining the association between respondents’ comfort levels with AI in health care, the use of personal health data for AI with and without consent, and key factors including sociodemographics, digital health literacy, and health conditions. Nonresponse weighted and unweighted models (Tables 4–6 in Multimedia Appendix 1) yielded results similar to the fully weighted analyses, supporting the sensitivity and robustness of the findings.

**Table 3.** Ordinal regression results (fully weighted): associations between AI comfort levels, use of personal health data, and sociodemographic factors, digital health literacy, and health conditions in 2023 Canadian Digital Health Survey.

	Model 1: Comfort level with the use of AI in health care		Model 2: Comfort level with the use of personal health data in AI with consent		Model 3: Comfort level with the use of personal health data in AI without consent	
Predictor and category	OR (95% CI)	*P* value	OR (95% CI)	*P* value	OR (95% CI)	*P* value
Age group (years) (ref=16‐24)						
25‐34	1.00 (0.80‐1.25)	.99	0.83 (0.67‐1.04)	.10	0.83 (0.68‐1.03)	.09
35‐54	0.91 (0.74‐1.12)	.35	0.72 (0.59‐0.89)	.00	0.73 (0.60‐0.88)	.00
55‐64	1.02 (0.82‐1.28)	.84	0.93 (0.74‐1.15)	.49	0.77 (0.63‐0.95)	.01
65+	1.47 (1.17‐1.84)	<.001	1.22 (0.97‐1.54)	.09	0.96 (0.78‐1.20)	.74
Sex (ref=female)						
Male	1.50 (1.36‐1.65)	<.001	1.39 (1.27‐1.53)	<.001	1.56 (1.42‐1.71)	<.001
Household income (ref=60,000‐100,000)						
>100,000	1.21 (1.08‐1.37)	<.001	1.16 (1.03‐1.30)	.01	1.05 (0.94‐1.18)	.37
<60,000	0.87 (0.77‐0.99)	.03	0.83 (0.74‐0.95)	.01	0.86 (0.76‐0.97)	.02
Citizenship (ref=citizen)						
Noncitizen	1.49 (1.18‐1.89)	<.001	1.20 (0.96‐1.49)	.11	1.28 (1.02‐1.61)	.03
Race (ref=Asian)						
Black/African origin	0.96 (0.71‐1.28)	.76	0.78 (0.59‐1.02)	.07	0.71 (0.54‐0.94)	.02
Other	0.78 (0.61‐1.00)	.05	0.77 (0.62‐0.97)	.03	0.83 (0.67‐1.04)	.11
White	0.77 (0.66‐0.89)	<.001	0.78 (0.68‐0.90)	<.001	0.69 (0.60‐0.80)	<.001
Education (ref=college level)						
Graduate college and higher	1.29 (1.15‐1.44)	<.001	1.25 (1.12‐1.40)	<.001	1.08 (0.97‐1.21)	.15
High school	0.90 (0.77‐1.05)	.17	0.89 (0.76‐1.03)	.11	0.90 (0.78‐1.04)	.15
<High school	0.88 (0.62‐1.23)	.44	1.08 (0.76‐1.53)	.66	0.75 (0.54‐1.05)	.09
Employment Status (Ref= Employed)						
Unemployed	1.01 (0.88‐1.14)	.94	1.06 (0.93‐1.21)	.38	0.89 (0.78‐1.01)	.07
Digital health literacy	1.06 (1.05‐1.07)	<.001	1.05 (1.04‐1.06)	<.001	1.04 (1.03‐1.05)	<.001
Number of chronic conditions	1.04 (1.00‐1.08)	.04	1.07 (1.03‐1.11)	.00	1.03 (0.99‐1.07)	.12

Age showed significant association with comfort levels with AI use in health care. In Model 1, older adults aged 65+ years exhibited higher odds of greater comfort with AI in health care (OR 1.47, 95% CI 1.17‐1.84; *P*<.001) compared to respondents aged 16‐24 years. In Model 2, respondents aged 35‐54 years (OR 0.72, 95% CI 0.59‐0.89; *P*<.001) exhibited lower odds of comfort when personal health data were used in AI with consent, while other age groups showed no statistically significant difference. In Model 3, comfort declined further among respondents aged 35‐54 years (OR 0.73, 95% CI 0.60‐0.88; *P*<.001) and 55‐64 years (OR 0.77, 95% CI 0.63‐0.95; *P*=.01) when personal health data were used in AI without consent, suggesting greater sensitivity to consent among middle-aged adults.

Sex was a consistent predictor across all three models, with men exhibiting higher odds of greater comfort of AI use in health care than women (Model 1: OR 1.50, 95% CI 1.36‐1.65; *P*<.001; Model 2: OR 1.39, 95% CI 1.27‐1.53; *P*<.001; Model 3: OR 1.56, 95% CI 1.42‐1.71; *P*<.001), indicating that men consistently report greater comfort with AI use in health when personal health data were used irrespective of consent.

Respondents with higher annual household incomes (above CAD 100,000) were significantly more likely to be comfortable with AI use in health care (OR 1.21, 95% CI 1.08‐1.37; *P*<.001) and with the use of personal data with consent (OR 1.16, 95% CI 1.03‐1.30; *P*=.01) when compared to those earning between CAD 60,000 and 100,000. Further, lower-income respondents (CAD <60,000) consistently reported lower comfort levels across all three models (OR range 0.83‐0.87, *P*<.05), suggesting that financial disparities may influence attitudes toward AI in health care.

We also found citizenship status in Canada to be a significant predictor in 2 out of 3 models. Noncitizens exhibited higher odds of comfort with AI use in health care (OR 1.49, 95% CI 1.18‐1.89; *P*<.001) and when personal health data were used without consent (OR 1.28, 95% CI 1.02‐1.61; *P*=.03), though the association was not significant in the with-consent model (OR 1.20, 95% CI 0.96‐1.49; *P*=.11). Overall, these findings suggest that noncitizens may perceive AI applications in health care more positively than citizens and are likely to exhibit more comfort level in personal health data being used for AI applications in healthcare irrespective of the consent.

Compared with Asian-origin respondents, White respondents exhibited lower odds of comfort across all models (OR range=0.69‐0.78, *P*<.001). Those identifying as “Other” racial groups also had lower odds in Models 1 and 2 (OR range=0.77‐0.78, *P*<.05). For Black or African-origin respondents, the association was significant only in Model 3 (OR 0.71, 95% CI 0.54‐0.94; *P*=.02), indicating reduced comfort when personal health data were used without consent.

Our analysis also showed higher educational attainment to be positively associated with comfort with AI use in health care and when personal health data were used with consent. Respondents with graduate-level or more education were significantly more comfortable (Model 1: OR 1.29, 95% CI 1.15‐1.44; *P*<.001; Model 2: OR 1.25, 95% CI 1.12‐1.40; *P*<.001). Those with only a high school education or lower did not show significant differences compared to the reference group (college-level education).

Digital health literacy emerged as a strong and consistent predictor across all three models. Each one-unit increase in digital literacy was associated with a 4%‐6% increase in odds of greater comfort (Model 1: OR 1.06, 95% CI 1.05‐1.07; *P*<.001; Model 2: OR 1.05, 95% CI 1.04‐1.06; *P*<.001; Model 3: OR 1.04, 95% CI 1.03‐1.05; *P*<.001), indicating that individuals with greater proficiency in using digital health tools were more comfortable with AI use in health care, both in general health care settings and when personal health data were involved.

The number of chronic health conditions was positively associated with comfort in Models 1 (OR 1.04, 95% CI 1.00‐1.08; *P*=.04) and 2 (OR 1.07, 95% CI 1.03‐1.11; *P*<.001) but not in Model 3 (OR 1.03, 95% CI 0.99‐1.07; *P*=.12), suggesting that individuals with multiple chronic illnesses were more comfortable with AI use in health care, particularly when personal data were used with consent. Employment status was not significantly associated with AI comfort in any of the three models.

## Discussion

### Principal Findings

To our knowledge, this is one of the first few studies to examine public attitudes toward use of AI in health care, with specific focus on the influence of sociodemographic, digital health literacy, and health-related factors. Overall, study respondents reported mixed levels of knowledge about AI and a considerable proportion (42.39%) expressing discomfort with AI use in health care. When personal health data were used for AI solutions with consent, the proportion of individuals becoming comfortable with AI use increased (64.69%), and when AI applications used personal deidentified health data without consent, a higher proportion (52.61%) expressed discomfort. A relatively higher proportion of respondents expressed greater comfort when AI was used in nonclinical areas like tracking epidemics and for improving healthcare workflows.

We found significant variations in knowledge levels of AI and comfort levels pertaining to AI use in health care based on sociodemographic, digital literacy, and the number of health conditions. Our results indicate that men, noncitizens, higher-income respondents, and respondents with greater digital health literacy exhibited higher odds of reporting comfort with AI use in health care and with the use of personal health data for AI. Older adults (65+ y) demonstrated higher comfort with AI use in health care, while younger (25–34 y) and middle-aged (35–54 y) adults were less comfortable with AI using their personal data, especially without consent.

Compared to Asian-origin respondents, White and Other racial groups had significantly lower comfort levels across models, while Black or African-origin respondents were notably less comfortable, only when personal health data were used for AI applications without consent. This finding suggests that lower comfort among Black respondents is not a general discomfort with AI but rather a heightened sensitivity to nonconsensual data use, thus underscoring the critical importance of transparency and opt-in data policies to foster trust among minority groups. Our findings also indicated that noncitizens exhibited higher comfort levels with AI use in health care as compared to Canadian citizens. The observed higher comfort among Asian-origin and noncitizen respondents and lower comfort among Black respondents suggests that broader cultural or experiential factors may influence attitudes toward AI in health care. Future qualitative or mixed-methods studies are needed to explore these factors. Prior research has shown that historical experiences, prior exposure to technology [33], and privacy concerns shape levels of trust in health technologies, particularly among minority populations [34,35]. Additionally, there is also some evidence that suggests that willingness to share personal health data for AI use depends strongly on the institution collecting the data and its intended purpose [24].

While digital health literacy improved comfort levels with use of AI, we also found that individuals with multiple health conditions were more accepting of AI when personal health data use was consensual. Individuals with multiple chronic conditions may have greater familiarity with varied health technologies and tools like wearables due to their increased prevalence [36-39], promoting appreciation for AI’s potential in managing complex conditions.

### Implications

One of the critical challenges in deploying AI solutions in health care is ensuring fairness and reducing algorithmic bias, which often arises from unrepresentative training datasets [40-42]. To build AI models that produce accurate, equitable, and generalizable outcomes, they must be trained on large, diverse, and high-quality datasets that reflect the complete range of patient demographics and health conditions [43]. Our findings point to the importance of placing explicit patient consent at the core of all efforts in developing AI solutions.

Health care institutions and policymakers must establish standardized protocols for obtaining patient consent for AI use, ensuring that data collection aligns with ethical and legal frameworks (eg, HIPAA [Health Insurance Portability and Accountability Act] in USA, PIPEDA [Personal Information Protection and Electronic Documents Act] in Canada, and GDPR [General Data Protection Regulation] in Europe) [44,45]. These policies must define whether patient data is being used for research, commercial development, or clinical decision-making. They must also clarify how long the data will be stored, who can access it, and whether patients have the right to withdraw consent at any time [46]. Without well-defined guidelines, the risk of unauthorized data usage and breaches increases, undermining public confidence in health care AI.

Even when patients provide consent, strong privacy protections must be in place, particularly when data are pooled across multiple health systems. There is a growing concern about deidentification and whether anonymized health data can still be reidentified using advanced AI techniques [47]. To mitigate these risks, health care institutions must implement privacy-preserving solutions, such as federated learning, where AI models are trained across decentralized data sources without transferring raw patient data [48,49]. Blockchain-based consent management can offer a secure way for patients to track and manage their data access, while strict data governance frameworks are essential to ensure AI developers use health data more responsibly.

This study shows that comfort with AI in health care is strongly influenced by sociodemographic factors and digital literacy. Individuals who trust AI and understand how it works are more likely to support AI-driven health care applications, while those with privacy concerns or lower digital literacy may resist AI use in health care, specifically when personal health data is used without consent. Addressing these concerns through educational initiatives, transparent policies, and patient engagement strategies can help build public confidence in AI solutions in health care.

Our findings also indicate that respondents were significantly less comfortable with AI use when personal health data were used without explicit consent. This highlights a crucial ethical dilemma—even if deidentified, patient data still carries risks if used without oversight or patient involvement. Future research and policy discussions should explore how much control patients should have over their deidentified data, what level of transparency AI developers must provide to patients, and how AI models trained on patient data should be evaluated for fairness and accountability. Algorithmic bias, as seen in lower comfort among certain racial groups like African Americans, especially when consent is absent, could exacerbate health care disparities if AI models are trained on unrepresentative datasets [41]. Mitigating algorithmic bias through diverse dataset inclusion and continuous performance monitoring can help reduce disparities [50,51]. Accountability in clinical decision-making is critical to ensure that AI supports, rather than overrides, clinician judgment, while prioritizing patient autonomy in data use strengthens trust [52,53]. These ethical challenges highlight the need for aligning AI deployment with patient expectations and equitable outcomes.

To build trustworthy AI-enabled health care solutions, policymakers and health administrators need to design targeted public awareness campaigns, co-developed with patient advocates, that clearly explain AI’s clinical role and how it uses patient data [54]. In addition, implementing opt-in consent policies [55,56] can help alleviate patient fears about misuse of their data. Culturally tailored digital literacy programs can also help boost patient confidence about AI use in health care. Beyond patient engagement, standardized bias audits for all clinical AI tools [52], while establishing patient review boards or advisory committees to gather patient feedback and assess ethical implications before deployment can be effective [57].

### Limitations

This study has several important limitations. First, the cross-sectional design captures public attitudes at a single point in time. The survey was done at the end of 2023 in Canada, when new generational AI technologies were still emerging. As advances in AI technologies and public awareness evolve, attitudes may change, making longitudinal studies necessary. Second, the self-reported nature of the data may introduce bias. Respondents could have misestimated their AI knowledge and comfort levels due to social desirability. Additionally, self-reported digital literacy may not accurately reflect actual proficiency in digital health technologies. Third, the study explored respondent attitudes toward AI, without assessing whether they had any prior exposure to any AI health care tools, such as chatbots, etc. Fourth, though we had a fairly large sample size, it may not be representative of the general population, restricting the generalizability of results. Specifically, our sample included relatively small proportions of noncitizens (4.68%) and Black or African-origin respondents (3.66%), which reduces statistical power for these subgroups. Consequently, subgroup findings should be interpreted with caution. Fifth, our operationalization of chronic health conditions as a composite count is a measurement limitation. Different health conditions, based on their nature and severity, could influence varied levels of technological use and engagement. Future research could explore how specific health conditions, and their nature and severity, influence attitudes toward AI. We also acknowledge possible nonresponse bias, as over 3226 cases were removed due to missing answers on AI-related questions. This reduction in analytic sample size may have excluded individuals with systematically different attitudes toward AI. Although weighting adjustments were used to correct for this, some bias may remain, as the weighting cannot account for unobserved factors. For instance, respondents who systematically avoided answering AI-related questions may hold strong, unmeasured attitudes such as anxiety or mistrust toward AI, which could bias our analytic sample. Finally, while the study examined broad sociodemographic and health factors, it did not delve into more specific determinants of AI trust, such as ethical concerns, data security apprehensions, or past experiences with health care technologies. Future research could explore these in greater detail to better understand the specific reasons behind patient acceptance of AI in health care. Additional investigation through qualitative or mixed-method studies could throw light on specific nuances that shape the patient attitudes toward AI use in health care.

### Conclusions

In conclusion, this study documents moderate levels of knowledge and comfort levels of the public regarding AI use in health care. Further, it highlights how sociodemographic characteristics, digital literacy, and health conditions are associated with public knowledge and comfort levels regarding AI use in health care. Our findings suggest significant socioeconomic disparities around the comfort levels with AI use in health care, while concerns persist around AI use without patient consent. These findings highlight the importance of transparent policies, patient education, and ethical data governance to improve public trust in AI-driven health care.

## Supplementary material

10.2196/77501 Multimedia Appendix 1 Sensitivity analysis: comparison of unweighted, IPW-weighted, and fully weighted ordinal logistic regression results.

## Abbreviations

AI: artificial intelligence
CDHS: Canadian Digital Health Survey
GDPR: General Data Protection Regulation
HIPAA: Health Insurance Portability and Accountability Act
IPW: inverse probability weight
OR: odds ratio
PIPEDA: Personal Information Protection and Electronic Documents Act
