# Supplementary material for: Listening to Patients’ Voices on the Use of AI in Health Care: Cross-Sectional Study
Source: J Med Internet Res. 2025 Dec 5;27:e77501. doi: 10.2196/77501 (PMC12680129; doi:10.2196/77501)
Supplement: Multimedia Appendix 1 [file jmir-v27-e77501-s001.docx]

**Table 4. Sensitivity Analysis: Comparison of Unweighted, IPW-Weighted, and Fully Weighted Ordinal Logistic Regression Results for Comfort with AI Use in Healthcare**

|  |  | **Unweighted** | | **Nonresponse-Adjusted Weighted (Svy IPW)** | | **Fully Weighted**  **(Svy IPW * CDHS weights)** | |
| --- | --- | --- | --- | --- | --- | --- | --- |
| **Variable** | **Category** | **OR (95% CI)** | **p** | **OR (95% CI)** | **p** | **OR (95% CI)** | **p** |
| Age group (ref = 16–24) | 25–34 | 0.99 (0.81–1.20) | 0.91 | 0.98 (0.81–1.19) | 0.84 | 1.00 (0.80–1.25) | 0.99 |
|  | 35–54 | 0.93 (0.78–1.12) | 0.43 | 0.91 (0.76–1.09) | 0.29 | 0.91 (0.74–1.12) | 0.35 |
|  | 55–64 | 1.06 (0.87–1.29) | 0.57 | 1.03 (0.85–1.25) | 0.78 | 1.02 (0.82–1.28) | 0.84 |
|  | 65+ | 1.50 (1.23–1.83) | p < .0001 | 1.47 (1.21–1.80) | p < .0001 | 1.47 (1.17–1.84) | 0.001 |
| Sex (ref = Female) | Male | 1.43 (1.31–1.57) | p < .0001 | 1.48 (1.35–1.63) | p < .0001 | 1.50 (1.36–1.65) | p < .0001 |
| Household income (ref = $60–100 K) | >100 K | 1.20 (1.08–1.34) | 0.001 | 1.20 (1.07–1.33) | 0.001 | 1.21 (1.08–1.37) | 0.002 |
|  | <60 K | 0.83 (0.74–0.92) | 0.001 | 0.85 (0.75–0.95) | 0.006 | 0.87 (0.77–0.99) | 0.031 |
| Citizenship (ref = Citizen) | Non-citizen | 1.43 (1.15–1.79) | 0.002 | 1.48 (1.18–1.85) | 0.001 | 1.49 (1.18–1.89) | 0.001 |
| Race (ref = Asian) | Black/African origin | 0.96 (0.74–1.24) | 0.73 | 0.94 (0.72–1.23) | 0.65 | 0.96 (0.71–1.28) | 0.76 |
|  | Other | 0.70 (0.58–0.86) | p < .0001 | 0.74 (0.59–0.92) | 0.007 | 0.78 (0.61–1.00) | 0.046 |
|  | White | 0.76 (0.66–0.87) | p < .0001 | 0.77 (0.67–0.88) | p < .0001 | 0.77 (0.66–0.89) | p < .0001 |
| Education (ref = College level) | Graduate College + | 1.29 (1.17–1.43) | p < .0001 | 1.28 (1.16–1.42) | p < .0001 | 1.29 (1.15–1.44) | p < .0001 |
|  | High school | 0.94 (0.82–1.07) | 0.34 | 0.92 (0.80–1.05) | 0.21 | 0.90 (0.77–1.05) | 0.17 |
|  | < High school | 1.00 (0.74–1.35) | 1.00 | 0.97 (0.71–1.32) | 0.83 | 0.88 (0.62–1.23) | 0.44 |
| Employment (ref = Employed) | Unemployed | 1.03 (0.92–1.15) | 0.62 | 1.01 (0.90–1.14) | 0.81 | 1.01 (0.88–1.14) | 0.94 |
| Digital health literacy |  | 1.06 (1.05–1.07) | p < .0001 | 1.06 (1.05–1.07) | p < .0001 | 1.06 (1.05–1.07) | p < .0001 |
| Number of Chronic Conditions |  | 1.04 (1.01–1.08) | 0.02 | 1.04 (1.01–1.08) | 0.02 | 1.04 (1.00–1.08) | 0.04 |

Note. OR = odds ratio; CI = confidence interval.

**Table 5. Sensitivity Analysis: Comparison of Unweighted, IPW‑Weighted, and Fully Weighted Ordinal Logistic Regression Results for Comfort with Use of Personal Health Data for AI with Consent**

|  |  | **Unweighted** | | **Nonresponse-Adjusted Weighted (Svy IPW)** | | **Fully Weighted**  **(Svy IPW * CDHS weights)** | |
| --- | --- | --- | --- | --- | --- | --- | --- |
| **Variable** | **Category** | **OR (95% CI)** | **p** | **OR (95% CI)** | **p** | **Svy OR (95% CI)** | **p** |
| Age group (ref = 16–24) | 25–34 | 0.80 (0.66–0.98) | 0.03 | 0.81 (0.66–0.98) | 0.03 | 0.83 (0.67–1.04) | 0.10 |
|  | 35–54 | 0.73 (0.61–0.88) | 0.001 | 0.72 (0.60–0.87) | 0.001 | 0.72 (0.59–0.89) | 0.002 |
|  | 55–64 | 0.95 (0.78–1.16) | 0.64 | 0.94 (0.77–1.15) | 0.54 | 0.93 (0.74–1.15) | 0.49 |
|  | 65+ | 1.29 (1.06–1.57) | 0.01 | 1.28 (1.04–1.57) | 0.02 | 1.22 (0.97–1.53) | 0.09 |
| Sex (ref = Female) | Male | 1.33 (1.21–1.45) | p < .0001 | 1.36 (1.24–1.49) | p < .0001 | 1.39 (1.27–1.53) | p < .0001 |
| Household income (ref = $60–100 K) | >100 K | 1.18 (1.06–1.31) | 0.003 | 1.16 (1.05–1.30) | 0.006 | 1.16 (1.03–1.30) | 0.013 |
|  | <60 K | 0.81 (0.72–0.91) | p < .0001 | 0.82 (0.73–0.93) | 0.001 | 0.83 (0.74–0.95) | 0.005 |
| Citizenship (ref = Citizen) | Non-citizen | 1.25 (1.01–1.55) | 0.04 | 1.27 (1.04–1.56) | 0.02 | 1.20 (0.96–1.49) | 0.11 |
| Race (ref = Asian) | Black/African origin | 0.84 (0.65–1.08) | 0.18 | 0.84 (0.65–1.07) | 0.16 | 0.78 (0.59–1.02) | 0.07 |
|  | Other | 0.68 (0.56–0.82) | p < .0001 | 0.71 (0.58–0.88) | 0.001 | 0.77 (0.62–0.97) | 0.025 |
|  | White | 0.76 (0.66–0.87) | p < .0001 | 0.77 (0.68–0.87) | p < .0001 | 0.78 (0.68–0.90) | p < .0001 |
| Education (ref = College level) | Graduate College + | 1.21 (1.09–1.34) | p < .0001 | 1.22 (1.10–1.35) | p < .0001 | 1.25 (1.12–1.40) | p < .0001 |
|  | High school | 0.91 (0.80–1.03) | 0.13 | 0.90 (0.78–1.03) | 0.12 | 0.89 (0.76–1.03) | 0.11 |
|  | < High school | 1.10 (0.81–1.48) | 0.56 | 1.11 (0.80–1.53) | 0.54 | 1.08 (0.76–1.53) | 0.66 |
| Employment (ref = Employed) | Unemployed | 1.04 (0.93–1.17) | 0.49 | 1.03 (0.92–1.17) | 0.58 | 1.06 (0.93–1.21) | 0.38 |
| Digital health literacy |  | 1.05 (1.04–1.05) | p < .0001 | 1.05 (1.04–1.06) | p < .0001 | 1.05 (1.04–1.06) | p < .0001 |
| Number of Chronic Conditions |  | 1.07 (1.03–1.11) | p < .0001 | 1.07 (1.03–1.11) | p < .0001 | 1.07 (1.03–1.11) | 0.001 |

Note. OR = odds ratio; CI = confidence interval.

**Table 6. Sensitivity Analysis: Comparison of Unweighted, IPW‑Weighted, and Fully Weighted Ordinal Logistic Regression Results for Comfort with Use of Personal Health Data for AI without Consent**

|  |  | **Unweighted** | | **Nonresponse-Adjusted Weighted (Svy IPW)** | | **Fully Weighted**  **(Svy IPW * CDHS weights)** | |
| --- | --- | --- | --- | --- | --- | --- | --- |
| **Variable** | **Category** | **OR (95% CI)** | **p** | **OR (95% CI)** | **p** | **OR (95% CI)** | **p** |
| Age group (ref = 16–24) | 25–34 | 0.91 (0.75–1.10) | 0.34 | 0.89 (0.74–1.06) | 0.19 | 0.83 (0.68–1.03) | 0.09 |
|  | 35–54 | 0.81 (0.68–0.97) | 0.02 | 0.78 (0.66–0.92) | 0.003 | 0.73 (0.60–0.89) | 0.001 |
|  | 55–64 | 0.86 (0.71–1.04) | 0.11 | 0.82 (0.68–0.98) | 0.03 | 0.77 (0.63–0.95) | 0.01 |
|  | 65+ | 1.03 (0.85–1.25) | 0.78 | 1.00 (0.83–1.21) | 0.99 | 0.96 (0.78–1.19) | 0.74 |
| Sex (ref = Female) | Male | 1.51 (1.38–1.65) | p < .0001 | 1.55 (1.42–1.70) | p < .0001 | 1.56 (1.42–1.71) | p < .0001 |
| Household income (ref = $60–100 K) | >100 K | 1.05 (0.94–1.16) | 0.41 | 1.03 (0.93–1.15) | 0.55 | 1.05 (0.94–1.18) | 0.37 |
|  | <60 K | 0.84 (0.75–0.94) | 0.002 | 0.84 (0.75–0.94) | 0.002 | 0.86 (0.76–0.97) | 0.02 |
| Citizenship (ref = Citizen) | Non-citizen | 1.29 (1.05–1.60) | 0.02 | 1.30 (1.06–1.59) | 0.01 | 1.28 (1.02–1.61) | 0.03 |
| Race (ref = Asian) | Black/African origin | 0.70 (0.55–0.90) | 0.006 | 0.73 (0.57–0.93) | 0.01 | 0.71 (0.54–0.94) | 0.02 |
|  | Other | 0.71 (0.59–0.86) | p < .0001 | 0.77 (0.63–0.95) | 0.01 | 0.83 (0.67–1.04) | 0.11 |
|  | White | 0.69 (0.61–0.79) | p < .0001 | 0.70 (0.62–0.79) | p < .0001 | 0.69 (0.60–0.80) | p < .0001 |
| Education (ref = College level) | Graduate College + | 1.10 (1.00–1.22) | 0.05 | 1.09 (0.99–1.21) | 0.08 | 1.08 (0.97–1.21) | 0.15 |
|  | High school | 0.98 (0.86–1.11) | 0.71 | 0.94 (0.83–1.08) | 0.39 | 0.90 (0.78–1.04) | 0.15 |
|  | < High school | 0.90 (0.67–1.21) | 0.49 | 0.85 (0.62–1.15) | 0.28 | 0.75 (0.54–1.05) | 0.09 |
| Employment (ref = Employed) | Unemployed | 0.92 (0.82–1.02) | 0.12 | 0.89 (0.79–1.01) | 0.06 | 0.89 (0.78–1.01) | 0.07 |
| Digital health literacy |  | 1.04 (1.03–1.05) | p < .0001 | 1.04 (1.03–1.05) | p < .0001 | 1.04 (1.03–1.05) | p < .0001 |
| Number of Chronic Conditions |  | 1.02 (0.99–1.06) | 0.25 | 1.03 (0.99–1.06) | 0.15 | 1.03 (0.99–1.07) | 0.12 |

Note. OR = odds ratio; CI = confidence interval..
